# Quantitative association analysis between PM_2.5_ concentration and factors on industry, energy, agriculture, and transportation

**DOI:** 10.1038/s41598-018-27771-w

**Published:** 2018-06-21

**Authors:** Nan Zhang, Hong Huang, Xiaoli Duan, Jinlong Zhao, Boni Su

**Affiliations:** 1 Department of Mechanical Engineering, The University of Hong Kong, Hong Kong, China; 2 Institute of Public Safety Research, Department of Engineering Physics, Tsinghua University, Beijing, China; 3 School of Energy and Environmental Engineering, University of Science and Technology Beijing, Beijing, China; 4 Electric Power Planning & Engineering Institute, Beijing, China

## Abstract

Rapid urbanization is causing serious PM_2.5_ (particulate matter ≤2.5 μm) pollution in China. However, the impacts of human activities (including industrial production, energy production, agriculture, and transportation) on PM_2.5_ concentrations have not been thoroughly studied. In this study, we obtained a regression formula for PM_2.5_ concentration based on more than 1 million PM_2.5_ recorded values and data from meteorology, industrial production, energy production, agriculture, and transportation for 31 provinces of mainland China between January 2013 and May 2017. We used stepwise regression to process 49 factors that influence PM_2.5_ concentration, and obtained the 10 primary influencing factors. Data of PM_2.5_ concentration and 10 factors from June to December, 2017 was used to verify the robustness of the model. Excluding meteorological factors, production of natural gas, industrial boilers, and ore production have the highest association with PM_2.5_ concentration, while nuclear power generation is the most positive factor in decreasing PM_2.5_ concentration. Tianjin, Beijing, and Hebei provinces are the most vulnerable to high PM_2.5_ concentrations caused by industrial production, energy production, agriculture, and transportation (IEAT).

## Introduction

PM_2.5_ (particulate matter ≤2.5 μm) has recently emerged as a serious pollutant in many countries. Some Asian countries such as India^1^, Japan^2^, and Malaysia^3^ have severe PM_2.5_ pollution problems. The situation has been very challenging in China^4,5^. China is experiencing extremely rapid urbanization which leads to high risk of PM_2.5_ pollution. This problem has attracted wide attention since 2013^6^. In Beijing, the capital of China, the highest daily average PM_2.5_ concentration measured was more than 500 μg/m^3^, which is 20-fold higher than the WHO guideline value^7^. Similar high concentrations have occurred several times in the past four years. According to a report from the Asian-development Bank, less than 1% of the 500 largest cities in China meet the WHO air quality guideline values (10 μg/m^3^ for the annual mean and 25 μg/m^3^ for the daily mean)^8^. Long-term exposure to PM_2.5_ has been consistently linked to heart and lung disease^9^, and reduces personal resistance^10^. For every PM_2.5_ concentration increase of 10 μg/m^3^, the risk of emergency hospital admissions for cerebrovascular diseases increased by 1.29%^11^. An aggressive global program of PM_2.5_ mitigation in line with WHO guidelines could avoid 750,000 (23%) of the current 3.2 million associated deaths per year^12^. Ways to efficiently control PM_2.5_ under rapid urbanization and aging populations need to urgently be found.

The Chinese government has placed great emphasis on PM_2.5_ control. The Chinese State Council released the ‘Atmospheric Pollution Prevention and Control Action Plan’ on September 10, 2013 which aimed to reduce PM_2.5_ by up to 25% by 2017 relative to the 2012 level^13^. At an APEC meeting in October 2014, Chinese President Xi Jinping stated that China will make a concerted effort to reduce air pollution^14^. In the 13^th^ Five-Year Plan (2016–2020), environmental pollution control is one of the first tasks specified. The plan also advocates the use of ‘big’ data to support pollution mitigation^15^.

Satellite and ground monitoring data are usually used to predict PM_2.5_ in the short term^16,17^. Models such as the WRF-Chem model^18^, the GEOS-Chem model^19^, and the CMAQ model^20^ are also used to analyze PM_2.5_ concentrations. Many studies have been devoted to finding the influencing factors for PM_2.5_ concentration. Meteorological factors such as temperature^21^, wind speed^22^, and rainfall^23^, and human factors such as industrial processes^24^, energy production and consumption^25^, and transportation^26^ are the most important. Previous research has shown that many human activities severely impact air pollution such as crop straw burning^27^, coal burning^28^, and vehicle exhaust emissions^29^. Many research has been carried out on PM_2.5_ concentration using Chemistry Transport Models (CTMs). CTMs can simulate the distribution of PM_2.5_ concentration in certain regions. Currently, however, this method still has some limitations. First, more work should be done on the mechanism of PM_2.5_ formation and development^30^. Second, data on pollution sources in a whole nation are often not accurate enough and will lead to significant simulation error^31^.

In this paper, PM_2.5_ concentration is studied through a different approach. We tried to figure out the most important influencing factors of industrial production, energy production, agriculture, and transportation (IEAT) on PM_2.5_ concentration based on available statistics data. Based on millions of collected PM_2.5_ data, we determined the spatial and temporal characteristics of PM_2.5_ distribution in mainland China, and analyzed trends in PM_2.5_ for different provinces. We also collected multisource statistical data that included meteorological and IEAT-factors for each month from January 2013 to May 2017. We found associations between PM_2.5_ and IEAT-factors, and developed a regression formula PM_2.5_ concentration based on 10 primary factors. In addition, we calculated the meteorological and IEAT contributions to PM_2.5_ levels in the different provinces. The results are helpful for governments providing macroscopic mitigation plans for controlling PM_2.5_ in the future.

## Data and Methods

### Data source

We summarized the collected data into 49 influencing factors, as listed in Table S1. These factors can be grouped into 5 categories: meteorology, industrial production, energy production, agriculture, and transportation. Since 2013, PM_2.5_ data were collected hourly by 391 monitoring stations managed by the Ministry of Environment Protection, China (MEP); PM_2.5_ data from January 2014 through May 2017 were collected daily by 190 monitoring cities from Air Quality Inspection Platform of China^32^; meteorological data between 2013 and 2016 were collected by 195 weather stations of the China Meteorological Administration (CMA)^33^; meteorological data between January and May 2017 are from the National Oceanic and Atmospheric Administration (NOAA)^34^; all data for industrial and energy production are from the National Bureau of Statistics of China (NBSC)^35^; all transportation data are from the Ministry of Transport, China (MOT)^36^; data for straw burning are from the Ministry of Environmental Protection, China (MEP)^37^; data for geographic division of mainland China are from the Ministry of Civil Affairs, China (MCA)^38^.

### Data processing

All data have different temporal collection frequency. PM_2.5_ and meteorological data were collected hourly and daily, while other data for human factors were collected monthly. To unify these data sets, the monthly value for each factor is used in this study, therefore a monthly averaged value is calculated for PM_2.5_ and meteorological data. PM_2.5_ and meteorological data, which were obtained by stations or cities, are averaged by province to obtain a provincial value.

To obtain the regression formula, all data are from the 53 months between January 2013 and May 2017. There are 34 provinces in China. Since there is no available data for Hong Kong, Macau, and Taiwan, we only considered the 31 provinces of mainland China. Therefore, the total data volume for each index should be 1643 (31 × 53) if no data was missing. In reality, there are data missing as shown in Table S1. We used linear interpolation to obtain this missing data. Most of the meteorological factors, and some of the human factors vary periodically by year. We therefore give priority to linear interpolation by year rather than by month. For example, if the data (X, M, Y) for province X in month M, Year Y is missing, we determine the data by linear interpolation based on data (X, M, Y − 1) and data (X, M, Y + 1). If data (X, M, Y − 1) or data (X, M, Y + 1) is missing, we use data (X, M − 1, Y) and data (X, M + 1, Y) to interpolate the missing data. We do not fill data outside the periods for which they were measured, for example, if data was only recorded from May 2015 to May 2017, then data before May 2015 cannot be determined via interpolation. Finally, 3.1% of the data was determined using linear interpolation.

To remove the differences due to geometrical area variation among the 31 provinces and the number of days in a month, all accumulative data (industrial production, energy production, agriculture, and transportation) were divided by province area and number of days in the month.

In this study, we used stepwise regression^39,40^ to process data to obtain the primary influencing factors that contribute the most to PM_2.5_ concentration. In statistics, stepwise regression is a method of fitting regression models in which the choice of predictive variables is made by an automatic procedure^41^. The variables ending up in the final equation signify the best combination of independent variables for predicting the dependent variable^42^. Stepwise regression is frequently used in the statistical analysis of air pollution and has the advantage of being able to avoid collinearity^43^. SPSS software (IBM SPSS, version 20) was used for the statistical analysis^44^.

All the 49 influencing factors (as listed in Table S1) are used as input variables. Using stepwise regression, 10 factors having the highest impact on PM_2.5_ are found. In addition, the regression formula for PM_2.5_ concentration is obtained.

## Results

### General analysis for PM_2.5_ in China

**Figure 1:**
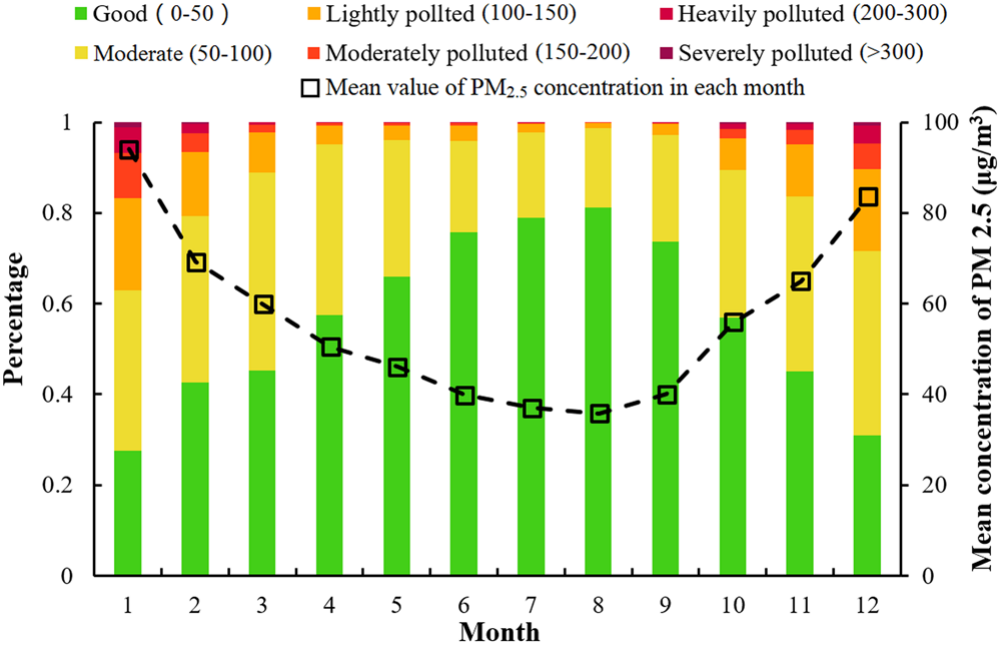
Pollution level analysis for 12 months (average value by 31 provinces) (data from January 1^st^, 2013 to December 31^th^, 2016; unit: μg/m^3^).

Because of periodicity of meteorological impacts and human behavior, PM_2.5_ variation is also periodic. Figure 1 shows the pollution level and mean concentration of PM_2.5_ in China for 12 months based on data from the past four years. The month with the best air quality is August, with a mean concentration of PM_2.5_ of 36.0 μg/m^3^. 81.1% of the days had good air quality, and 17.5% of the days had moderate air quality. January has the worst air quality with on average 94.3 μg/m^3^ of PM_2.5_. In January, only 27.6% of the days have good air quality, and 37.1% days have light, moderate, heavy, or severe pollution. Generally speaking, concentrations of PM_2.5_ are much higher in winter than in summer. Therefore, better control of PM_2.5_ in winter is critical.

**Figure 2:**
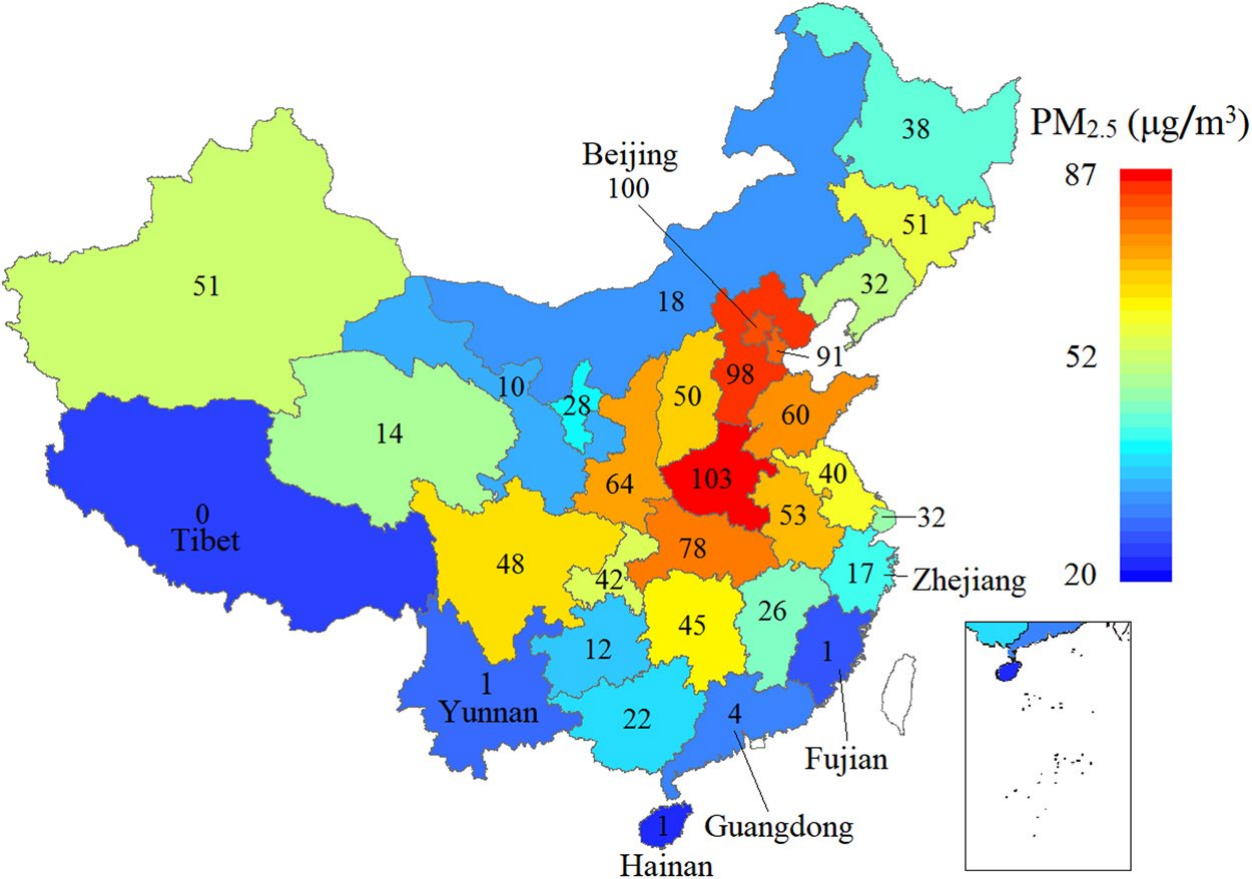
PM_2.5_ concentration distribution and number of yearly polluted days in 31 provinces of China (data from January 1^st^ 2013 to December 31^th^ 2016). (The picture was generated by ArcGIS 10.0, https://www.esri.com/en-us/home).

Figure 1 shows the temporal distribution of PM_2.5_, while Fig. 2 shows its spatial distribution. The number in Fig. 2 shows the average number of polluted days (PM_2.5_ concentration >100 μg/m^3^) per year. From Fig. 2, Henan, Hebei, Beijing, Tianjin, and Hubei ranked in the top five for average concentration of PM_2.5_. Beijing experiences about 100 polluted days on average every year. The average PM_2.5_ concentration in Beijing was 79.1 μg/m^3^ for the past 4 years. The five provinces with best air quality are Hainan, Tibet, Fujian, Yunnan, and Guangdong. Hainan has on average 20.0 μg/m^3^ of PM_2.5_ while there was not one polluted day in Tibet from 2013 to 2016. Generally, the highly polluted areas are North, Central and some parts of East China. The Coastal province and Northwest China have better air quality.

**Figure 3:**
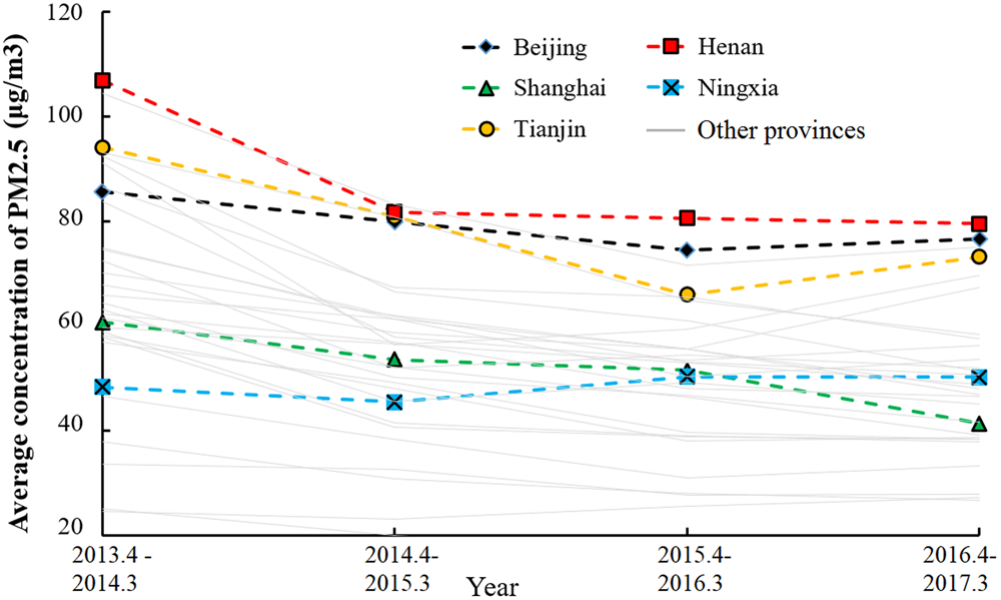
Concentration trends of PM_2.5_ in Beijing, Shanghai, Tianjin, Henan, and Ningxia provinces (data from April 1^st^ 2013 to March 31^th^ 2017).

Combining the results of spatial and temporal distribution of PM_2.5_, Fig. 3 shows the concentration of PM_2.5_ in five typical provinces from April 2013 to March 2017. Beijing (black dashed line) experiences serious air pollution during these years. The PM_2.5_ concentration in Beijing decreased between 2013 and 2015, but increased slightly again to 76.7 μg/m^3^ in 2016. Shanghai, with the highest income per capita in China, experienced a gradual decrease in PM_2.5_ concentration from 60.8 to 41.4 μg/m^3^ between April 2013 and March 2017. Henan, the province with the highest population, experienced the most serious air pollution in China. The PM_2.5_ concentration in Henan gradually decreased from 107.0 to 79.5 μg/m^3^ in the same period. Because of the impact of heavy industry, the PM_2.5_ concentration in Tianjin increased by 11.0% year-on-year. Ningxia, a representative city with low economic development, saw only a slight increase in PM_2.5_ concentration for the period covered by the data.

### Association between PM_2.5_ concentration and IEAT-factors

The ten most important factors influencing PM_2.5_ concentration, determined by stepwise regression, are listed in Table 1. Meteorological factors including temperature, air pressure, and wind speed are the most strongly associated with PM_2.5_ concentration. The top six IEAT factors having a strong association with PM_2.5_ concentration include the production of: natural gas; industrial boilers; ore; tractors; nuclear power, and locomotives.

**Table 1:** Top 10 influencing factors highly associated with PM_2.5_ concentration.

Rank	Factor	Coefficient	Standard error	Unit	Sig.	Accumu-lative R^2^
1	Temperature	−1.57 × 10^0^	7 × 10^−2^	μg·m^−3^·°C^−1^	0.000	0.358
2	Air pressure	8.3 × 10^−2^	6 × 10^−3^	μg·m^−3^·hPa^−1^	0.000	0.518
3	Wind speed	−1.2 × 10^1^	1 × 10^0^	μg·m^−3^·m^−1^·s	0.000	0.577
4	Natural gas production	4.3 × 10^2^	5 × 10^1^	10^−4^ μg·m^−3^·m^−3^·km^2^·d	0.000	0.634
5	Industrial boiler production	6.0 × 10^−1^	9 × 10^−2^	μg·m^−3^·t (vapor) ^−1^·km^2^·d	0.000	0.673
6	Ore production	3.0 × 10^0^	4 × 10^−1^	μg·m^−3^·t^−1^·km^2^·d	0.000	0.694
7	Tractor production	1.1 × 10^3^	2 × 10^2^	10^4^ μg·m^−3^·km^2^·d	0.000	0.707
8	Nuclear power generation	−7 × 10^1^	2 × 10^1^	10^−4^ μg·m^−3^·kW·h·km^2^·d	0.000	0.713
9	Locomotive production	2.4 × 10^0^	8 × 10^−1^	10^4^ μg·m^−3^·km^2^·d	0.003	0.717
10	24-hour rainfall	−6 × 10^−1^	2 × 10^−1^	μg·m^−3^·mm^−1^	0.004	0.721

Constant = 1.37 × 10^1^ μg·m^−3^.

Shaanxi, Tianjin, and Beijing have the highest production of natural gas per unit area. Henan, Shanghai, and Jiangsu are the three biggest industrial boiler producers per unit area. Hebei, Beijing, and Liaoning have the highest production of ore per unit area. Shandong, Henan, and Chongqing are the top three provinces in tractor production per unit area. Zhejiang, Tianjin, and Beijing rank highest in the production of locomotives per unit area. Nuclear power generation is the only positive human factor among the top ten factors. Using clean energy can efficiently reduce PM_2.5_ air pollution. Just 7 out of 31 provinces have nuclear power generation. The average PM_2.5_ concentrations per year in Zhejiang, Guangdong, and Fujian provinces (which have high nuclear power generation) are 49.5, 37.2, and 30.4 μg/m^3^, respectively. Constant in this formula is 13.7 which means the background value of PM_2.5_ concentration is 13.7 μg/m^3^ in China. In addition, R^2^ of the linear fitting by these 10 factors is 0.721.

According to values in Table 1, the regression formula for PM_2.5_ concentration can be written as (Eq. 1):

1 $$\begin{array}{c}{C}_{{PM}_{2.5}}=-\,1.57T+8.3\times {10}^{-2}{P}_{A}-12{S}_{W}-0.6{R}_{24}+4.3\times {10}^{2}{P}_{NG}+0.60{P}_{IB}\\ \,\,\,\,+3.0{P}_{O}+1.1\times {10}^{3}{P}_{T}-7\times 10{P}_{NP}+2.4{P}_{RL}+13.7\end{array}$$

where ${C}_{{PM}_{2.5}}$ is the PM_2.5_ concentration (μg/m^3^), *T* average temperature (°C), *P*_*A*_ average air pressure (hPa), *S*_*W*_ average wind speed (m·s^−1^), *R*_24_ average rainfall for 24 hours (mm); *P*_*NG*_, *P*_*IB*_, *P*_*O*_, *P*_*T*_, *P*_*NP*_, and *P*_*RL*_ are production rates (production per square kilometer per day) of natural gas (10^4^ m^3^·km^−2^·d^−1^), industrial boilers (t(vapor)·km^−2^·d^−1^), ore (t·km^−2^·d^−1^), tractors (km^−2^·d^−1^), nuclear power (10^4^ kW·h·km^−2^·d^−1^), and locomotives (10^−4^ km^−2^·d^−1^) per square kilometer per day, respectively.

### Meteorological and IEAT contributions to PM_2.5_ concentration

In this study, we divided the influencing factors into meteorological and IEAT elements. Meteorological and IEAT contributions to PM_2.5_ concentration are calculated using Eq. 1 with meteorological and human factors, respectively.

**Figure 4:**
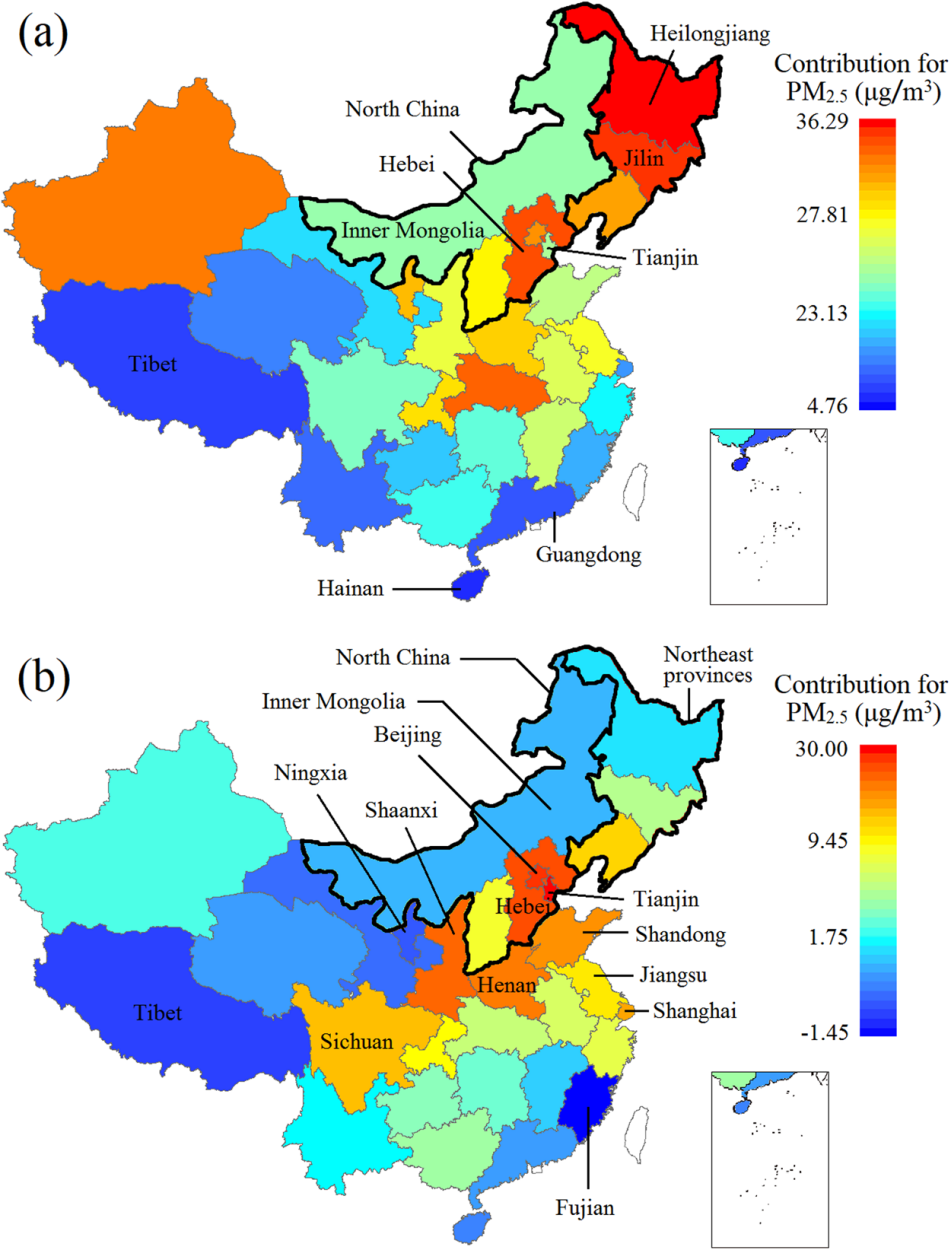
Contribution map for PM_2.5_. (**a**) Meteorological contribution. (**b**) IEAT contribution. (The picture was generated by ArcGIS 10.0, https://www.esri.com/en-us/home).

Figure 4(a,b) show the meteorological and IEAT contributions to PM_2.5_. In Fig. 4(a) we see that the three northeastern provinces of China and North China (except Inner Mongolia and Tianjin) have a high meteorological contribution to PM_2.5_, while the south of China has a lower meteorological contribution to PM_2.5_.

Heilongjiang (38.0 μg/m^3^), Jilin (36.3 μg/m^3^), and Hebei (33.5 μg/m^3^) provinces have the highest meteorological contribution, while Hainan (4.8 μg/m^3^), Tibet (14.0 μg/m^3^), and Guangdong (14.0) provinces have the lowest meteorological contribution. In Fig. 4(b) we see that the three northeastern provinces of China are not bad in regard to IEAT contribution to PM_2.5_. North China (except Inner Mongolia), Sichuan, Shaanxi, Henan, and Shandong provinces are high IEAT contributors to PM_2.5_. Some southern coastal provinces such as Shanghai and Jiangsu also have high IEAT contributions. Tianjin (30.5 μg/m^3^), Beijing (27.5 μg/m^3^), and Hebei province (26.7 μg/m^3^) have the highest IEAT contribution to PM_2.5_ while Fujian (−1.5 μg/m^3^), Tibet (0.0 μg/m^3^), and Ningxia (0.2 μg/m^3^) rank lowest. More detailed information can be found in Table S2.

### Verification

**Figure 5:**
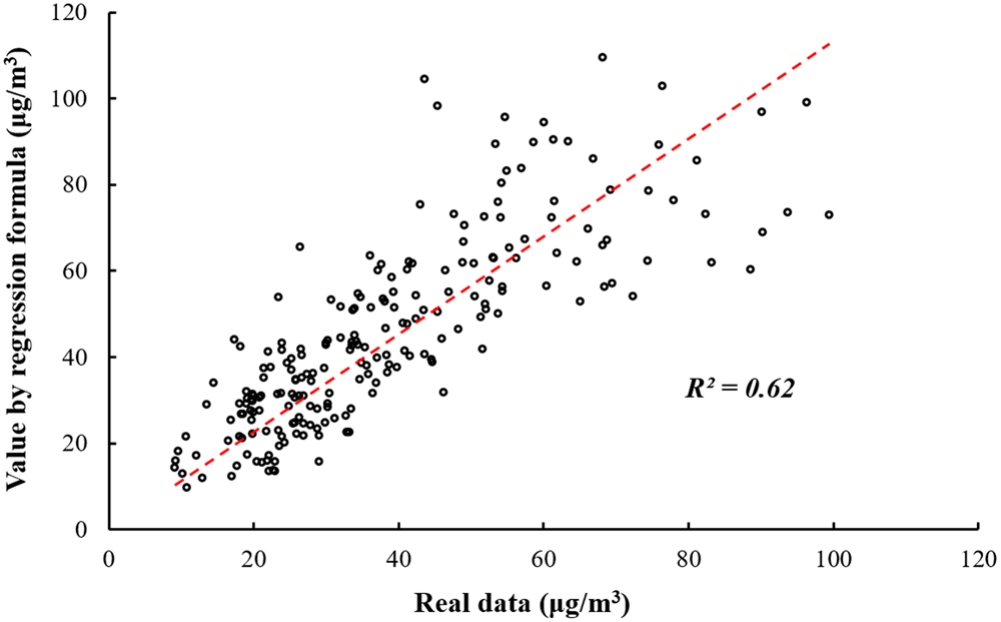
Comparison between the measured data of PM_2.5_ concentration (from June 2017 to December, 2017) and the values obtained by regression formula (Eq. 1).

Another group of data from June 2017 to December 2017 was used to verify the robustness of regression formula obtained above (Eq. 1). In Fig. 5, a total of 217 points show the monthly averaged PM_2.5_ data for 7 months of 31 provinces. From comparison between the measured data and calculated values by regression formula based on ten influencing factors from June 2017 to December 2017 (Fig. 5), R^2^ is 0.62, which is slightly lower than regression formula’s accumulative R^2^ (0.72). The average errors between the measured data and the calculated value is 11.1 μg/m^3^_._

## Discussion

In this study we investigated 49 influencing factors including meteorology, industrial production, energy production, agriculture, and transportation on PM_2.5_ concentrations. We determined a quantitative association between PM_2.5_ concentration and IEAT factors, and obtained a formula for PM_2.5_ concentration considering 10 primary factors based on stepwise regression. Stepwise regression is used because it is suitable for processing collinear data. We have tried to use common linear regression to process data, and found that the results are not as good as results obtained by stepwise regression (see *Supplementary Information* for details).

In this study, we analyzed PM_2.5_ concentrations in China from January 2013 to May 2017. The average PM_2.5_ concentration over 12 months shows an upward parabola. Severe pollution (>300 μg/m^3^) appeared in winter in many areas of China. Using clean energy such as nuclear power to replace coal burning power plants is a very efficient way to reduce the number of severely polluted days in China. North and Central China have serious PM_2.5_ pollution problems. Research has shown that a 10 μg/m^3^ increase over a previous day’s PM2.5 level results in a 1.78% increase in respiratory related mortality and a 1.03% increase in stroke related mortality^45^. In China, residents in high-PM_2.5_ concentration areas look forward to gale force winds to reduce pollution. However, reducing PM_2.5_ generated by human activity is the key solution. One approach would be a reasonable distribution of PM_2.5_ sources to help balance PM_2.5_ concentration between highly-populated areas and rural areas. In addition, efforts should be made to reduce the number of severely polluted days rather than just reducing the average PM_2.5_ concentration.

Meteorological contributions to PM_2.5_ are high in the three northeastern provinces of China and North China (excluding Inner Mongolia and Tianjin). These areas are cold in winter, resulting in more coal consumption. Less rainfall in these inland areas is another meteorological reason for high PM_2.5_ levels. North China (excluding Inner Mongolia), Central China, and some provinces of East China have high IEAT contributions to PM_2.5_. Beijing is a typical polluted city which had an average PM_2.5_ concentration of 76.7 μg/m^3^ from April 2016 to March 2017. In the 13th Five-Year Plan, The Ministry of Environment Protection, China (MEP) set a target for the concentration of PM_2.5_ in Beijing to be reduced to 56 μg/m^3^ by 2020. This is a considerable challenge. Shanghai provides a good example where in the past four years, the PM_2.5_ concentration has gradually decreased even though urban construction continues and the economy keeps improving.

According to stepwise regression, we found that of the 49 influencing factors (meteorology, industrial production, energy production, agriculture, and transportation), the production of natural gas, industrial boilers, ore, tractors, nuclear power, and locomotives are the top six IEAT factors contributing to PM_2.5_ concentrations. Since the production of natural gas, energy, ore, locomotives and tractors do not need to be concentrated in one area, future planning should include spreading these industries over wider areas to avoid creating areas with high population densities and heavy pollution. Finally, the regression formula was verified by the data from another seven months from June 2017 to December 2017.

Some researchers are focusing on the relationship between human factors and PM_2.5_ pollution. Previous research has suggested that clean fuels such as natural gas should replace the coal used for small domestic boilers to reduce air pollution^46^ because combustion for natural gas is cleaner. However, some recent studies noticed that several air pollutants, including VOCs, NO_x_, PM_2.5_ and SO_2_, will be emitted during production stage of the natural gas^47–49^. Our results also show the production of natural gas is strongly related to PM_2.5_ concentrations. Using clean energy (such as solar power, wind power, and nuclear power) to replace fossil energy, rather than using natural gas to replace coal, would be a better solution for PM_2.5_ control. Industrial boilers, which are usually used for burning coal^50^, are bad for air quality^51^. We also found that provinces with high production of industrial boilers, usually have high PM_2.5_ concentrations. Industrial boilers are usually heavy and not convenient for long-range transportation, so most of the produced boilers are likely to be installed and used locally, leading to this phenomenon. Through analysis of collected 24-h PM_2.5_ samples in Brazil, researchers found that environmental contamination is led by ore mining and related activities such as the transport of products to and from the mines^52^. In our study, we found that production of ironstone and phosphate ore were the third biggest generators of PM_2.5_ (P < 0.001), followed by the production of natural gas and industrial boilers. Turkish researchers found that the PM_2.5_ concentration around tractors can reach thousands μg/m^3 ^^53^. We found that the production of tractors is also strongly related to PM_2.5_ concentration (P < 0.001). New tractors are usually transported to nearby regions, and tractor operation will also generate PM_2.5_. In New York, the levels of PM_2.5_ rapidly reached a peak when a diesel-powered locomotive passed^54^. In China, locomotives are widely used. We found that producing locomotives will increase PM_2.5_ concentration. High production of locomotives generally reflects a high level of heavy industry, which lead to serious air pollution. As for meteorological factors, many studies have shown that the PM_2.5_ concentration is negatively correlated with wind speed and rainfall^55–58^ which agrees with our findings. Because many human factors such as coal burning are strongly related to meteorological factors, the impacts of meteorological factors on PM_2.5_ concentration are difficult to quantitatively measure.

There are some limitations to this study. Because of data limitation, we considered only some influencing factors in meteorology, industrial production, energy production, agriculture, and transportation. Other meteorological and IEAT factors may also influence PM_2.5_ levels. For example, energy consumption is very important on emissions and air pollution. However, accurate monthly fuel consumption data at provincial level is unavailable, mainly because there are numerous distributed consumers. In the future, we should take these factors into consideration, if advanced statistical method is developed and accurate energy consumption data are available. In addition, some unconsidered human factors are strongly related to meteorological factors. For example, the consumption of coal and fireworks is high when the temperature is low (Spring Festival which consumes many fireworks is around January and February)^59^. Therefore, the meteorological contribution in this study is mainly comprised of both unconsidered human and meteorological factors. In addition, all data is from January 2013 to May 2017. The precision could be improved if we could collect more data over longer periods. We used the province as the spatial unit in this study. City-scale data should be analyzed in the future once more precise data is obtained. In our data processing, some data was obtained using yearly and monthly linear interpolation, and this may slightly influence the precision of the regression formula for PM_2.5_ concentration. Moreover, since all factors and results are related to industrial technologies, personal living habits, and a few other characteristics, the association should be adjusted when being used for analysis of PM_2.5_ concentrations in other countries.

## Conclusions

PM_2.5_ in China in spatial and temporal dimensions was analyzed for data from January 2013 to May 2017. We quantitatively obtained the impacts of meteorology, industrial production, energy production, agriculture, and transportation on PM_2.5_ concentration over an extended period. We found that production of natural gas, industrial boilers, ore production, tractors, and locomotives were the five human factors with the strongest association with PM_2.5_ concentration. The model and the results provide efficient references for governments to make better plans on controlling PM_2.5_ concentrations.

In the future, more types of data, longer time periods, and more detailed regionalization should be considered to improve the precision of the association analysis for PM_2.5_ concentration.

## Electronic supplementary material

Supplementary Information
