# Supplementary material for: Quantitative association analysis between PM2.5 concentration and factors on industry, energy, agriculture, and transportation
Source: Sci Rep. 2018 Jun 21;8:9461. doi: 10.1038/s41598-018-27771-w (PMC6013430; doi:10.1038/s41598-018-27771-w)
Supplement: Supplementary file 1 — Supplementary Information [file 41598_2018_27771_MOESM1_ESM.docx]

Quantitative association analysis between PM_2.5_ concentration and factors on industry, energy, agriculture, and transportation

Nan Zhang ^1^, Hong Huang ^2^, Xiaoli Duan^3^, Jinlong Zhao ^2^, Boni Su ^4,*^

^1^ Department of Mechanical Engineering, The University of Hong Kong, Hong Kong SAR, China

^2^ Institute of Public Safety Research, Department of Engineering Physics, Tsinghua University, Beijing, China

^3^ School of Energy and Environmental Engineering, University of Science and Technology Beijing, Beijing, China

^4^ Electric Power Planning & Engineering Institute, Beijing, China

^*^ Corresponding author

bnsu@eppei.com

**Supplementary information**

**Table S1. Influencing factors for meteorological and IEAT-related factors**

| Category | Influencing factors | Unit | Components | Time Range | Data Volume |
| --- | --- | --- | --- | --- | --- |
| Meteorology (4) | Average air pressure | hPa | - | 2013 Jan - 2017 May | 1638 |
|  | Average temperature | ℃ | - | 2013 Jan - 2017 May | 1643 |
|  | Average wind speed | m·s^‑1^ | - | 2013 Jan - 2017 May | 1643 |
|  | Average 24-hour rainfall | mm | - | 2013 Jan - 2017 May | 1640 |
| Energy  Production (10) | Raw coal | t·km^-2^·d^-1^ | - | 2015 Mar - 2017 May | 689 |
|  | Crude oil | t·km^-2^·d^-1^ | - | 2013 Mar - 2017 May | 997 |
|  | Natural gas | 10^4^ m^3^·km^-2^·d^-1^ | - | 2013 Feb – 2017 May | 983 |
|  | Processed crude oil | t·km^-2^·d^-1^ | - | 2013 Mar - 2017 May | 1233 |
|  | Coke | t·km^-2^·d^-1^ | - | 2013 Feb - 2017 May | 1314 |
|  | Thermal power generation | 10^4^ kW·h·km^-2^·d^-1^ | - | 2013 Feb - 2017 May | 1394 |
|  | Hydropower generation | 10^4^ kW·h·km^-2^·d^-1^ | - | 2013 Feb - 2017 May | 1326 |
|  | Nuclear power generation | 10^4^ kW·h·km^-2^·d^-1^ | - | 2013 Feb - 2017 May | 506 |
|  | Wind power generation | 10^4^ kW·h·km^-2^·d^-1^ | - | 2013 Mar - 2017 May | 1411 |
|  | Coal gas | 10^4^ m^3^·km^-2^·d^-1^ | - | 2013 Feb - 2017 May | 1379 |
| Industrial Production (32) | Ore mining | t·km^-2^·d^-1^ | Ferrous ore | 2013 Mar - 2017 May | 1188 |
|  |  |  | Phosphate ore | 2013 Mar - 2017 May | 692 |
|  | Food | t·km^-2^·d^-1^ | Crude salt | 2013 Mar - 2017 May | 989 |
|  |  |  | Sugar | 2013 Mar - 2017 May | 753 |
|  |  |  | Beverage | 2013 Mar - 2017 May | 1333 |
|  | Textile | t·km^-2^·d^-1^ | Yarn | 2013 Mar - 2017 May | 1254 |
|  |  |  | Cloth | 2013 Mar - 2017 May | 1053 |
|  | Paper | t·km^-2^·d^-1^ | Machine-made paper & cardboard | 2013 Mar - 2017 May | 1289 |
|  |  |  | Newsprint | 2013 Mar - 2017 May | 730 |
|  | Chemical products | t·km^-2^·d^-1^ | Sulfuric acid (H_2_SO_4_) | 2013 Mar - 2017 May | 1235 |
|  |  |  | Caustic soda (NaOH) | 2013 Mar - 2017 May | 1232 |
|  |  |  | Soda (Na_2_CO_3_) | 2013 Mar - 2017 May | 987 |
|  |  |  | Agricultural fertilizer | 2013 Mar - 2017 May | 1262 |
|  |  |  | Chemical pesticide | 2013 Mar - 2017 May | 1093 |
|  |  |  | Ethylene (C_2_H_4_) | 2013 Mar - 2017 May | 811 |
|  |  |  | Plastic in primary form | 2013 Mar - 2017 May | 1297 |
|  |  |  | Synthetic detergent | 2013 Mar - 2017 May | 1098 |
|  |  |  | Chemical fiber | 2013 Mar - 2017 May | 1108 |
|  | Metallurgy | t·km^-2^·d^-1^ | Cast iron | 2013 Mar - 2017 May | 1228 |
|  |  |  | Crude steel | 2013 Mar - 2017 May | 1288 |
|  |  |  | Steel | 2013 Mar - 2017 May | 1282 |
|  |  |  | Ten nonferrous metals | 2013 Apr - 2017 May | 1221 |
|  |  |  | Aluminum oxide | 2013 Mar - 2017 May | 617 |
|  |  |  | Copper | 2013 Mar - 2017 May | 1191 |
|  |  |  | Aluminum | 2013 Mar - 2017 May | 1263 |
|  | Plastic product | t·km^-2^·d^-1^ | - | 2013 Mar - 2017 May | 1311 |
|  | Cement | t·km^-2^·d^-1^ | - | 2013 Mar - 2017 May | 1333 |
|  | Flat glass | weight case·km^-2^·d^-1^ | - | 2013 Mar - 2017 May | 1204 |
|  | Industrial boiler | t (vapor)·km^-2^·d^-1^ | - | 2013 Mar - 2017 May | 1162 |
|  | Engine | kW·km^-2^·d^-1^ | - | 2013 Mar - 2017 May | 1093 |
|  | Metal cutting machine tool | km^-2^·d^-1^ | - | 2013 Mar - 2017 May | 1192 |
|  | Electric hand tools | km^-2^·d^-1^ | - | 2013 Mar - 2017 May | 693 |
|  | Metallurgy equipment | 10^-4^ t·km^-2^·d^-1^ | - | 2013 Mar - 2017 May | 912 |
|  | Equipment for cement | 10^-4^ t·km^-2^·d^-1^ | - | 2013 Mar - 2017 May | 690 |
|  | Packaging equipment | 10^-4^ km^-2^·d^-1^ | - | 2013 Mar - 2017 May | 891 |
|  | Tractor | 10^-4^ km^-2^·d^-1^ | - | 2013 Mar - 2017 May | 1043 |
|  | Air pollution control equipment | 10^-4^ km^-2^·d^-1^ | - | 2013 Mar - 2017 May | 1028 |
|  | Rail locomotive | 10^-4^ km^-2^·d^-1^ | - | 2013 Mar - 2017 May | 459 |
|  | Automobile | 10^-4^ km^-2^·d^-1^ | - | 2013 Mar - 2017 May | 1203 |
|  | Civilian steel ship | t (load)·km^-2^·d^-1^ | - | 2013 Mar - 2017 May | 892 |
|  | Generating equipment | kW·km^-2^·d^-1^ | - | 2013 Mar - 2017 May | 1115 |
|  | Alternating current motor | kW·km^-2^·d^-1^ | - | 2013 Mar - 2017 May | 1120 |
|  | Household appliance | km^-2^·d^-1^ | Washing machine | 2013 Mar - 2017 May | 719 |
|  |  |  | Refrigerator | 2013 Mar - 2017 May | 781 |
|  |  |  | Air conditioner | 2013 Mar - 2017 May | 784 |
|  |  |  | Television | 2013 Mar - 2017 May | 885 |
|  | SPC exchange | km^-2^·d^-1^ | - | 2013 Mar - 2017 May | 623 |
|  | Fax | km^-2^·d^-1^ | - | 2013 Mar - 2017 May | 329 |
|  | Mobile communication base station equipment | channel· km^-2^·d^-1^ | - | 2013 Mar - 2017 May | 628 |
|  | Cell phone | km^-2^·d^-1^ | - | 2013 Mar - 2017 May | 908 |
|  | Microcomputer equipment | km^-2^·d^-1^ | - | 2013 Mar - 2017 May | 812 |
|  | Integrated circuit | km^-2^·d^-1^ | - | 2013 Mar - 2017 May | 904 |
|  | Electrical Instruments | km^-2^·d^-1^ | - | 2013 Mar - 2017 May | 1096 |
|  | Printing equipment | km^-2^·d^-1^ | - | 2013 Mar - 2017 May | 476 |
| Agriculture (1) | Incident of straw burning | 10^-4^ km^-2^·d^-1^ | - | 2014 Jun - 2017 May | 805 |
| Transportation (2) | Volume of highway passenger | (person·km)·km^-2^·d^-1^ | - | 2013 Jun - 2017 May | 1271 |
|  | Volume of highway cargo | (t·km)·km^-2^·d^-1^ | - | 2013 Jun - 2017 May | 1178 |

## Table S2. Meteorology-related and IEAT-related contribution to PM_2.5_ in 31 provinces (Meteor-C: meteorology-related contribution, IEAT-C: industrial production, energy, agriculture, and transportation-related contribution).

| Province | Meteor-C (ug/m^3^) | IEAT-C (ug/m^3^) | Province | Meteor-C (ug/m^3^) | IEAT-C (ug/m^3^) | Province | Meteor-C (ug/m^3^) | IEAT-C (ug/m^3^) |
| --- | --- | --- | --- | --- | --- | --- | --- | --- |
| Beijing | 31.0 | 27.5 | Hubei | 31.7 | 5.4 | Zhejiang | 22.8 | 8.8 |
| Tianjin | 25.2 | 30.5 | Hunan | 23.6 | 3.0 | Anhui | 26.4 | 7.8 |
| Hebei | 33.5 | 26.7 | Guangdong | 14.0 | 1.0 | Fujian | 21.0 | -1.5 |
| Shanxi | 28.7 | 9.1 | Guangxi | 23.5 | 3.3 | Jiangxi | 26.4 | 1.5 |
| Inner Mongolia | 24.7 | 1.1 | Hainan | 4.8 | 0.7 | Shandong | 25.7 | 20.6 |
| Liaoning | 30.2 | 10.6 | Chongqing | 28.8 | 9.7 | Shaanxi | 26.6 | 24.0 |
| Jilin | 36.3 | 3.6 | Sichuan | 24.2 | 12.3 | Gansu | 22.2 | 0.4 |
| Heilongjiang | 38.0 | 1.6 | Guizhou | 21.3 | 3.3 | Qinghai | 20.0 | 1.0 |
| Shanghai | 20.1 | 17.1 | Yunnan | 17.4 | 1.9 | Ningxia | 29.3 | 0.2 |
| Jiangsu | 27.0 | 10.0 | Tibet | 14.0 | 0.0 | Xinjiang | 31.2 | 2.2 |
| Henan | 29.0 | 23.8 |  |  |  |  |  |  |

**Comparison on two regression models on data processing**

We tried using common linear regression to process data. The regression formulas between PM_2.5_ concentration and each single factor are obtained. The top ten factors that PM_2.5_ concentration most linearly dependent on (factors with largest R^2^) are listed in Table S3.

**Table S3. Top ten influencing factors obtained using common linear regression.**

| Factor | Regression Formula | R^2^ |
| --- | --- | --- |
| Temperature | $C_{PM2.5}=-1.6T+73$ | 0.358 |
| 24-hour rainfall | $C_{PM2.5}=-2.7R_{24}+56$ | 0.159 |
| Industrial boiler production | $C_{PM2.5}=1.2P_{IB}+41$ | 0.118 |
| Natural gas production | $C_{PM2.5}=6.7{\times10}^{2}P_{NG}+41$ | 0.098 |
| Volume of highway cargo | $C_{PM2.5}=1.8{\times10}^{-3}V_{C}+39$ | 0.076 |
| Ore production | $C_{PM2.5}=5.1P_{O}+43$ | 0.074 |
| Air pressure | $C_{PM2.5}=6.2{\times10}^{-2}P_{A}-13$ | 0.060 |
| Tractor production | $C_{PM2.5}=2.1{\times10}^{3}P_{T}+43$ | 0.049 |
| Air pollution control equipment | $C_{PM2.5}=1.2{\times10}^{-1}P_{APC}+45$ | 0.045 |
| Incidents of straw burning | $C_{PM2.5}=5.3{\times10}^{1}I_{SB}+45$ | 0.040 |

There are differences between these ten factors and factors obtained using stepwise regression. Volume of highway cargo, air pollution control equipment production and incidents of straw burning are regarded as important factors obtained by common linear regression. While average wind speed, nuclear power generation and locomotive production are another important factors in top ten factors obtained by stepwise regression.

In order to determine which method is better, multidimensional linear regressions are carried out. With only top ten influencing factors obtained by common linear regression considered, the multidimensional regression formula for PM_2.5_ concentration is:

| $C_{PM2.5}=-1.62T-0.2R_{24}+0.4P_{IB}+4.5{\times10}^{2}P_{NG}+0V_{C}+3.5P_{O}+6.5{\times10}^{-2}P_{A}+1.6{\times10}^{3}P_{T}+1.5{\times10}^{-2}P_{APC}-6I_{SB}+3.7$ | (1) |
| --- | --- |

where *C_PM2.5_* is the PM_2.5_ concentration (μg/m^3^), *T* average temperature (℃), *R_24_* average rainfall for 24 hours (mm), *P_A_* average air pressure (hPa), *V_C_* volume of highway cargo (t·km·km^-2^·d^-1^), *I_SB_* incidents of straw burning per square kilometer per day (km^-2^·d^-1^); *P_IB_*, *P_NG_*, *P_O_*, *P_T_*, and *P_APC_* are production rates of industrial boilers (t(vapor)·km^-2^·d^-1^), natural gas (10^4^ m^3^·km^-2^·d^-1^), ore (t·km^-2^·d^-1^), tractors (km^-2^·d^-1^), and air pollution control equipment (10^-4^ km^-2^·d^-1^) per square kilometer per day, respectively.

With only top ten influencing factors obtained by stepwise linear regression considered, the multidimensional regression formula for PM_2.5_ concentration is:

| $C_{PM2.5}=-1.57T+8.3{\times10}^{-2}P_{A}-12S_{W}+4.3{\times10}^{2}P_{NG}+0.60P_{IB}+3.0P_{O}+1.1{\times10}^{3}P_{T}-7\times10P_{NP}+2.4P_{RL}-0.6R_{24}+13.7$ | (2) |
| --- | --- |

where *S_W_* is average wind speed (m·s^-1^); *P_NP_*, and *P_RL_* are production rates (production per square kilometer per day) of nuclear power (10^4^ kW·h·km^-2^·d^-1^), and locomotives (10^-4^ km^-2^·d^-1^) per square kilometer per day, respectively.

The R^2^ of formula (1) is 0.658, while R^2^ of formula (2) is 0.721. That means Formula (2) can represent PM_2.5_ concentration better than Formula (1). In addition, Formula (1) is abnormal in some aspects. First, the coefficient of *V_C_* (volume of highway cargo) is 0 (in other words, there is no significant difference between 0 and the coefficient), indicating that *V_C_* makes no contribution to Formula (1) and is probably not an important influencing factor. Second, the coefficient of *I_SB_* (incidents of straw burning) is negative, meaning that the more straw burning leading to lower PM_2.5_ concentration, which is obviously irrational.

These problems are probably caused by variable collinearity. Stepwise regression is frequently used in the statistical analysis of air pollution and has the advantage of being able to avoid collinearity. Therefore, stepwise regression is used in this paper.
